# Improving infant sleep safety via electronic health record communication: a randomized controlled trial

**DOI:** 10.1186/s12887-020-02369-2

**Published:** 2020-10-08

**Authors:** Ethan A. Canty, Benjamin N. Fogel, Erich K. Batra, Eric W. Schaefer, Jessica S. Beiler, Ian M. Paul

**Affiliations:** 1 Department of Pediatrics, Oregon Health & Science University, Portland, OR USA; 2 Department of Pediatrics, Penn State College of Medicine, HS83, 500 University Drive, Hershey, PA 17033 USA; 3 Family and Community Medicine, Penn State College of Medicine, Hershey, PA USA; 4 Public Health Sciences, Penn State College of Medicine, Hershey, PA USA

**Keywords:** SIDS, SUID, HER, Electronic health record, Patient portals, Photograph

## Abstract

**Background:**

With increased use of telehealth, interventions to improve infant sleep environments have not been explored. This study sought to assess the feasibility and efficacy of using electronic health record patient portals to transmit photographs of infant sleep between mothers and healthcare professionals as part of an intervention to promote sleep environments consistent with AAP guidelines.

**Methods:**

One hundred eighty-four mother-newborn dyads consented to participate in a randomized trial requiring patient portal registration within 1 month of delivery. We first assessed feasibility as measured by a) the proportion of consented mothers enrolling in the portal and b) maternal adherence to prompts to submit photographs of their infant sleeping to the research team through the patient portal. Intervention group mothers were prompted at 1 and 2 months; controls were prompted only at 2 months. Efficacy was determined via research assistant review of submitted photographs. These assistants were trained to detect sudden unexplained infant death risk factors utilizing AAP guidelines. Standardized feedback was returned to mothers through the patient portal. We used Fisher’s Exact test to assess group differences in guideline adherence at 2 months.

**Results:**

One hundred nine mothers (59%) enrolled in the patient portal and were randomized to intervention (*N* = 55) and control (*N* = 54) groups. 21 (38, 95% CI 25–52%) intervention group participants sent photographs at 1 month and received personalized feedback. Across both groups at 2 months, 40 (37, 95% CI 28–46%) sent photographs; 56% of intervention group participants who submitted photographs met all safe sleep criteria compared with 46% of controls (difference 0.10, 95% CI − 0.26 to 0.46, *p* = .75). Common reasons for guideline non-adherence were sleeping in a room without a caregiver (43%), loose bedding (15%) and objects (8%) on the sleep surface.

**Conclusions:**

Utilizing the patient portal to individualize safe infant sleep is possible, however, we encountered numerous barriers in this trial to assess its effects on promoting safe infant sleep. Photographs of infants sleeping showed substantial non-adherence to AAP guidelines, suggesting further needs for improvement to promote safe infant sleep practices.

**Trial registration:**

Name: Improving Infant Sleep Safety With the Electronic Health Record; Clinicaltrials.gov: NCT03662048; Date of Registration: September 7, 2018;

Data Sharing Statement: None

## Background

Sudden Infant Death Syndrome (SIDS) rates decreased substantially between the years 1983–2012, associated with public health campaigns like “Back to Sleep” [1]. Nonetheless over 3000 deaths still occur annually due to Sudden Unexpected Infant Deaths (SUID), a term that includes SIDS of unknown cause or accidental suffocation and strangulation in bed [2].

The majority of SUIDs occur in infants between the ages of one and 4 months, and 90% of all SUIDs occur prior to the age of 6 months [3]. Therefore, optimizing sleep environments from early infancy remains the best practice for preventing SUIDs [4–6]. Adherence to SUID prevention recommendations is generally lower among lower income, less educated, and minority families [7–12]. Unfortunately, adherence is generally also poor in wealthier populations. A recent study demonstrated that even when such mothers knew they are under video observation, they commonly fail to adhere to American Academy of Pediatrics (AAP)-endorsed infant sleep recommendations [13]. Similar findings were recently reported in Australia [14]. This suggests either a knowledge gap about recommendations, an inability to comply with them, or a lack of belief that the risk is significant for their child.

Mobile health interventions and messaging to mothers of infants have shown some success in improving supine infant sleep rates and avoiding soft bedding use, suggesting technology and social media may be a possible intervention for improving infant sleep safety [15, 16]. Use of patient portals, secure online websites that give families convenient access to electronic health record (EHR)-based personal health information from anywhere with an Internet connection, has become widespread in healthcare [17, 18], with research showing that transmission of wound photographs via the patient portal to be an acceptable alternative to in-person general surgery care [19]. Furthermore, use of patient portals has been shown to improve patient access to information, insight into clinical conditions and health, patient communication and continuity of care, and preventive health delivery [20, 21]. Recognizing its potential to deliver preventive guidance for SUID, we conducted a randomized clinical trial designed to test the feasibility and efficacy of using the patient portal to reduce unsafe infant sleep practices and improve adherence to the AAP SIDS guidelines [5]. For feasibility, we aimed to see if mothers would register for patient portal use during the newborn period and then send photographs of their infant sleeping when prompted. For intervention efficacy, we sought to evaluate whether provision of safe sleep feedback in response to transmitted photographs at infant age 1 month was associated with greater safe sleep guideline adherence at 2 months compared with those receiving no guidance at 1 month.

## Methods

### Participants

Mothers and their term newborns were recruited in person by research staff from a single maternity ward (Penn State Milton S. Hershey Medical Center, Hershey, Pennsylvania, USA) before hospital discharge. In obtaining informed consent, mothers were informed that the primary purpose of the study was “to see if we can use the patient portal to provide more individualized healthcare for you and your baby, and the way we are trying to test this idea is to see if we can use the patient portal to more personalize safe infant sleep care.” All mothers with newborns at this center receive information on SIDS prevention and education on safe sleep during their stay on the maternity unit using Pennsylvania Department of Health approved printed materials, and safe sleep is modeled by providers throughout the stay. In addition, mothers are directed to sign an acknowledgement statement that they have received, read and understand the educational materials per Pennsylvania’s Sudden Infant Death Syndrome Education and Prevention Act [22].

Enrollment began in October 2018 and was completed in June 2019. To be eligible for the study, infants needed to be ≥37 weeks’ gestation and singleton with planned follow-up at a Penn State Health practice for primary care visits. Additionally, only English-speaking mothers ≥18 years old with full email and internet access were eligible. Finally, to participate, mothers were required to have an electronic device that can take photographs to be transmitted through the online patient portal, which they agreed to access.

During enrollment, mothers had two options to enroll their newborn in the patient portal following local health system procedures. The first option required them to complete a form. One of the researchers faxed the form to the patient portal office, and the mother then would receive an email with a link to enroll their newborn in the patient portal. In the second option, mothers could enroll by either calling the patient portal office while in the hospital or when at their newborn’s outpatient visit. Mothers were given the phone number of the patient portal office if they decided to call to enroll.

The Penn State Health Patient Portal user interface allows parents to view their child’s health data. Parents, at the minimum, can view provider summaries from each visit, lab results, vaccinations, and vital signs. The patient portal also has a messaging service whereby parents may select the provider to send an e-message. The patient portal allows attachments to be sent, including images, between providers and portal users. While not fully utilized for this purpose today, the portal is capable of supporting two-way information sharing and communication between providers and families that could be used for health promotion.

The Human Subjects Protection Office of the Penn State College of Medicine approved this study and it was registered at http://www.clinicaltrials.gov prior to first participant’s enrollment (NCT03662048). The study adheres to CONSORT guidelines.

### Randomization and study groups

After consenting to the study and being provided with information on how to register for the patient portal, mothers were encouraged to create a patient portal account for their infant within a month of delivery. Participants were given one reminder call and email per week encouraging them to enroll by the 1 month deadline in order to continue in the study. Even if a parent had at least one child with a Penn State Patient Portal, parents were required to register their newborn for a patient portal via a link sent by email, as described above. Randomization occurred after an account was created, was stratified by parity at the time of delivery (0 vs. ≥1) with a 1:1 allocation ratio, and was performed using REDCap software. Participants who did not enroll in the patient portal within a month were considered lost-to-follow-up and were not randomized.

Following randomization, at 1 month, intervention group mothers were sent a message through the patient portal with instructions on how to take two photographs of their infant sleeping on each of their nighttime sleep surfaces and how to send the photographs through the portal to the research team. Participants were asked to take two photographs from two different angles to capture the entire sleep surface. Participants received a reminder message to transmit the photographs each week until 2 weeks after the initial message or the photographs were received. The photographs were analyzed by trained research assistants (EAC and JSB) following training by a content expert (EKB) based on a checklist of risk factors for SUID described in the AAP guidelines (Table 1). Inter-rater reliability had been planned, but because study recruitment difficulties resulted in a lower than expected sample size, we determined that this was unlikely to affect study outcomes. Personalized feedback based on standardized scripts from the checklist was generated by the study team detailing AAP recommendations and sent to the mothers through the patient portal within 7 business days. This feedback detailed which features of the infant sleep environment were adherent to the AAP guidelines as well as corrective guidance for features that were non-adherent. Controls were not contacted at 1 month. At infant age 2 months, mothers in both study groups were asked to submit photographs, and identical procedures were followed as described above for the intervention group at 1 month. All infants were expected to receive standard health maintenance visits with their primary care provider, and guidance given as part of this study was only meant to supplement usual care.

**Table 1:** SUID risk factor checklist used to evaluate photographs

Unsafe sleep risk identified in infant’s photograph	Infant sleep consistent with AAP guidelines
□ Not sleeping on own sleep surface	□ Sleeping on own sleep surface
□ Not sleeping on back	□ Sleeping on back
□ Soft sleep surface	□ Firm sleep surface
□ Soft object in sleep area □ Pillow □ Stuffed animals or toys □ Other loose objects in sleep area:	□ No loose objects in sleep area
□ Loose bedding in sleep area	□ No loose bedding in sleep area
□ Dangling cords or electrical wires in sleep area	□ No dangling cords or electrical wires in sleep area
□ Sleeping in car seat, stroller, swing, carrier, sling, or other □ Sleeping on couch, armchair □ Sleeping on bed designed for an adult or older child	□ Sleeping in crib, bassinette, portable crib, or play yard
□ Bumper pads attached to crib slats	□ No bumper pads
□ Sleep wedge/positioner on sleep surface	□ No sleep wedge/positioner on sleep surface
□ Covering of the face and head	□ Face and head without any covering

### Measures

#### Demographics and baseline data

We collected data regarding the pregnancy, delivery, and nursery course for each mother and newborn from the medical record. Family demographic information was collected via survey during enrollment.

#### Determination of final outcomes based on AAP criteria

Using the checklist in Table 1, we determined whether an infant met all of the AAP recommendations for sleeping environments at 1 and 2 months. For any item on the checklist that could not be assessed, we elected to not presume the infant was placed in an at-risk environment. Sleep room location was determined via subjective parental reporting when photographs were transmitted.

#### Sample size and analysis plan

The primary outcome for this study was the proportion of participants at 2 months who were classified as meeting all of the safe sleep criteria based upon AAP guidelines [5]. Based upon AAP guideline adherence found in our prior research [13], we anticipated that 50% of controls would have identifiable SUID risk factors on the submitted photographs compared with 25% from the intervention group. A total of 57 mother-infant dyads in each randomized group yielded 80% power to detect that difference for a test conducted at the 5% level of significance. To account for potential attrition after randomization, we planned to enroll an additional 16 dyads for a total sample size of 130. For the primary outcome of proportion meeting AAP safe sleep criteria at 2 months, we used a two-sided Fisher’s Exact test conducted at the 5% level of significance to test for statistically significant differences between randomized groups.

A secondary outcome, which assessed the feasibility of the study, was defined as the proportion of patients who provided photographs through the patient portal. The proportions providing photographs were calculated for the intervention group only at 1 month; proportions were calculated for both groups combined at 2 months since we expected no difference in adherence to photograph requests between groups. We calculated 95% confidence intervals (CIs) for these proportions using the exact binomial method.

## Results

**Fig. 1:**
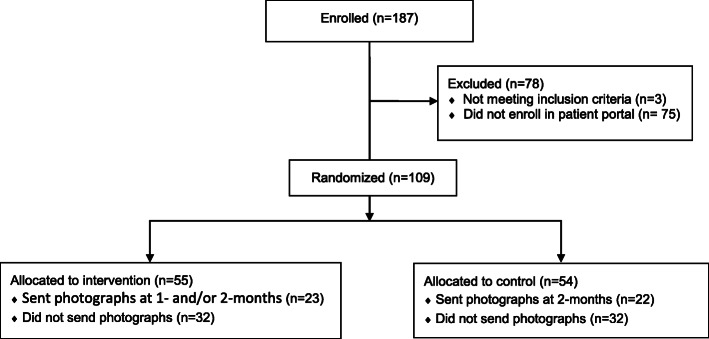
Consort Diagram

Among 187 participants who provided written informed consent, 184 (98%) mother-newborn dyads met all inclusion criteria and were asked to register their newborn for the patient portal. Only 109 (59%) enrolled their newborn in the patient portal and were randomized into intervention and control groups (Fig. 1). While this sample size of 109 is less than the 130 used in the study sample size calculation, recruitment was stopped to allow for timely completion of the trial.

Among the randomized cohort, mothers had a mean (SD) maternal age of 29.2 (4.9) years, were predominantly White, non-Hispanic, and in a dual-parent household (Table 2). The majority were college educated, had private insurance, were breastfeeding, and had household incomes greater than $50,000. Those mothers who enrolled, but did not fulfill criteria for randomization, namely did not enroll in the patient portal, were more likely to be racial/ethnic minorities, multiparous, single, Medicaid-insured, and smokers (*P* < .10 for each).

**Table 2:** Demographics of randomized cohort (*N* = 109)

	Intervention (*N* = 55)	Control (*N* = 54)
**Infant characteristics**		
**Female Sex, N (%)**	27 (49)	28 (52)
**Gestational age in weeks, mean (SD)**	39.4 (1.1)	39.4 (1.2)
**Birth weight in kg, mean (SD)**	3.38 (0.49)	3.37 (0.46)
**Feeding Mode, N (%)**		
Exclusive Breast	42 (76)	40 (74)
Exclusive Formula	5 (9)	7 (13)
Combination	8 (15)	7 (13)
**Maternal characteristics**		
**Mother’s age, Mean (SD)**	28.9 (4.0)	29.4 (5.7)
**Parity**		
0	27 (49)	25 (46)
≥ 1	28 (51)	29 (54)
**Smoke during pregnancy, N (%)**	7 (13)	5 (9)
**Mother’s Race, N (%)**		
Black	4 (7)	4 (7)
White	43 (78)	42 (78)
Asian	4 (7)	6 (11)
Other	4 (8)	2 (4)
**Hispanic or Latino Ethnicity, N (%)**	5 (9)	4 (7)
**Married or Cohabitating, N (%)**	50 (91)	45 (83)
**Insurance, N (%)**		
Private health insurance	45 (82)	42 (78)
Medicaid	10 (18)	10 (19)
Other	0 (0)	2 (4)
**Education, N (%)**		
High school graduate or less	12 (22)	11 (20)
Some college or technical school	15 (27)	13 (24)
Completed college	28 (51)	30 (56)
**Plan to work outside the home in next 12 months, N (%)**		
Yes	42 (76)	39 (72)
No	10 (18)	10 (19)
Unsure	3 (6)	5 (9)
**Number of people living in household, Median (IQR)**	2 (1–3)	2 (1–3)
**Household income, N (%)**		
< $50,000	15 (27)	12 (22)
$50,000 to <$100,000	18 (33)	23 (43)
≥ $100,000	15 (27)	13 (24)
Do not know or Refuse to Answer	7 (13)	6 (11)
**Type of home, N (%)**		
Single family	30 (55)	33 (61)
Multi-family	2 (4)	0 (0)
Apartment	9 (16)	9 (17)
Townhome	10 (18)	10 (19)
Mobile home or trailer	1 (2)	1 (2)
Other	3 (6)	1 (2)

### Feasibility

The effort required to enroll in the patient portal impeded randomization for the first 64 consenting participants. Mothers were required to undergo a time-sensitive three-step process to enroll their newborn in the patient portal, resulting in only one-third of this first group of consenting mothers being randomized. For the final 66 participants, we successfully streamlined the patient portal enrollment process after working with those managing this process to create a patient portal registration form. By the end of the trial, among the final 66 participants, two-thirds enrolled in the patient portal and were randomized.

The second part of the feasibility assessment involved the submission of photographs per the study protocol. The 55 intervention group mothers were sent e-messages at infant age 1 month with only 21 (38, 95% CI 25–52%) responding with photographs. At 2 months, 18 intervention group and 22 control group mothers sent photographs (37, 95% CI 28–46%). Following the first e-message by the study team, participants returned photographs through the patient portal at a median of 62 h (interquartile range (IQR) 37–70 h). The study team returned standardized feedback at a median of 24 h (IQR 13–51 h).

### Efficacy

#### Photograph characteristics

For the 55 participants in the intervention group, 32 (58%) never sent a photograph at either time point, 5 (9%) sent a photograph only at 1 month, 2 (4%) sent a photograph only at 2 months, and 16 (29%) sent a photograph at both time points. The response at 2 months was higher in the control group (41 to 33%), but not statistically significant (*P* = 0.39). At infant age 1 month (intervention group), those sending photographs were more likely to have completed a college degree. At infant age 2 months, for the entire cohort, those sending photographs were more likely to be white and have completed a college degree.

At infant age 1 month, intervention group mothers sent a median of 2 photographs (range 1–4; Table 3). For these 21 infants, 7 (33%) were not sleeping in a room with a caregiver, and the sleep location could not be determined in 3 (14%). For the sleep surface, the photographs showed non-AAP guideline adherent sleeping arrangements including those sleeping on non-firm surfaces (5%) and those with objects on their sleep surface (e.g. 10% had loose bedding, 5% had a facial covering). Accounting for all items on the checklist, 57% of intervention group infants met all safe sleep criteria at 1 month when including sleep location; 81% met all safe sleep criteria excluding sleep location.

**Table 3:** Infant sleep photograph characteristics

	Intervention - Month 1 (*N* = 21)	Intervention - Month 2 (*N* = 18)	Control - Month 2 (*N* = 22)
**Number submitted**			
Median (Range)	2 (1–4)	2 (2–4)	2 (1–4)
**Sleep location, N (%)**			
Own room	5 (24)	4 (22)	3 (14)
Parents’ room	11 (52)	7 (39)	16 (73)
Another room of the house	1 (5)	2 (11)	2 (9)
Sibling’s room	1 (5)	0 (0)	0 (0)
Cannot be determined	3 (14)	5 (28)	1 (5)
**Outermost piece of clothing, N (%)**			
Shirt	0 (0)	0 (0)	1 (5)
Shirt and pants	1 (5)	1 (6)	0 (0)
Onesy	1 (5)	2 (11)	5 (23)
Pajamas	5 (24)	2 (11)	4 (18)
Swaddle blanket	8 (38)	6 (33)	6 (27)
Sleep sack	6 (29)	5 (28)	4 (18)
Other	0 (0)	2 (11)	2 (9)
**Sleep surface, N (%)**			
Crib	9 (43)	8 (44)	7 (32)
Bassinet	5 (24)	5 (28)	10 (45)
Cradle	0 (0)	1 (6)	1 (5)
Bedside co-sleeper (“sidecar”)	0 (0)	0 (0)	1 (5)
Co-sleeper in middle of bed	0 (0)	0 (0)	1 (5)
Pac n Play, traveling bed, portable play yard	5 (24)	4 (22)	1 (5)
Rock n play	1 (5)	0 (0)	1 (5)
Other	1 (5)	0 (0)	0 (0)
**Firm sleep surface, N (%)**			
Yes	19 (90)	17 (94)	22 (100)
No	1 (5)	0 (0)	0 (0)
Unsure	1 (5)	1 (6)	0 (0)
**Sleep position, N (%)**			
Supine (on back)	21 (100)	17 (94)	21 (96)
Side	0 (0)	1 (6)	1 (5)
**Objects on sleep surface, N (%)**			
Yes (Infants with ≥1 object)	2 (10)	3 (17)	5 (23)
Pillow/cushion	0 (0)	0 (0)	2 (9)
Stuffed animal/pillow like toy	0 (0)	0 (0)	1 (5)
Loose bedding	2 (10)	2 (11)	4 (18)
Bumper pads	0 (0)	2 (11)	0 (0)
Sleep positioner/wedge	0 (0)	1 (6)	2 (9)
Loose cord/electrical wire	0 (0)	0 (0)	1 (5)
White Noise Machine	0 (0)	0 (0)	2 (9)
**Head covering, N (%)**	1 (5)	1 (6)	0 (0)
**Mobile/hanging toy within reach, N (%)**	2 (10)	1 (6)	0 (0)
**Sleep surface shared with another person, N (%)**	0 (0)	0 (0)	1 (5)
**Pacifier, N(%)**	2 (14)	2 (11)	1 (5)

At 2 months, among those sending photographs (*N* = 18), intervention group mothers sent a median of 2 (range 2–4). Sleep location (33% not in parent’s room), non-supine sleep (6%), and objects on the sleep surface were identified in some photographs (e.g. 11% with loose bedding, 11% with bumper pads, and 6% with a sleep wedge/position). Control group mothers sent a median of 2 photographs (range 1–4) at 2 months. For the 22 control infants with photographs, sleep location (23% not sleeping in parent’s room), bedsharing (5%), unapproved sleep surfaces, and objects on the sleep surface were identified (e.g.18% with loose bedding, 9% with pillows/cushions, 9% with a sleep positioner/wedge).

Comparing study groups at 2 months revealed no significant differences in adherence to safe sleep guidelines. 55.6% of intervention group participants who submitted photographs met all safe sleep criteria compared with 45.4% of controls (difference of 0.10, 95% CI − 0.26 to 0.46, *P* = 0.75) when sleep location was included. When excluding sleep location as a criteria, 83% of intervention group infants met all safe sleep criteria versus 68% of controls (difference of 0.15, 95% CI − 0.16 to 0.46, *P* = 0.46).

## Discussion

While it is possible to use the EHR to promote personalized sleep recommendations based on photographs submitted by mothers through the patient portal, this trial encountered numerous barriers that must be overcome for this prevention strategy to be implemented in clinical care. Our finding that mothers who submitted photographs demonstrated a high rate of non-adherence to AAP guidelines emphasizes that efforts to prevent sleep-related infant deaths must be enhanced, but the method we tested can only be used if enrollment in the patient portal is simple and mothers demonstrate a willingness to respond to prompts for photographs.

There have been few successful randomized clinical trials to improve infant safe sleep practices, but most focus on supine sleeping and bed sharing [15, 23, 24]. The rates of supine sleeping (95%) and lack of bed sharing (97.5%) attained in this study are much higher than have been attained in other studies, and demonstrate that those who sent photographs have sufficient knowledge about the importance of these two practices. In contrast, regarding adherence to the totality of the AAP guidelines, only 50% of participants met all safe sleep recommendations at age 2 months with no significant differences between study groups. This is disappointing as age 2 months is a time when cases of SUID are peaking and in need of innovative interventions to enhance preventive efforts as well as adherence to the AAP guidelines. Further, because the photographs are a glimpse at one point during an infant’s sleep, it is uncertain the extent to which these already low percentages decrease over the course of the night. Our previous study found that infants that changed locations overnight typically were moved to a sleep environment with a greater number of risk factors for SUID [13].

Enrolling in the patient portal impeded randomization for some of the initial participants who consented for the trial. After simplification of the procedures required to enroll, we were able to improve newborn patient portal enrollment rates. Our experience suggests a streamlined patient portal registration process can improve patient portal registration among patients and increase utilization. Additionally, efforts aimed at decreasing barriers for mothers with fewer resources and more barriers may need extra considerations to promote utilization.

Participant photograph submission rates were also lower than expected suggesting additional barriers to our approach. We have several hypotheses for this observation. The first 2 months of an infant’s life require extensive time from mothers, and communicating with a research team through a patient portal may not have been a priority. It is possible that rates may have been improved had such communication been initiated by primary care providers. Next, the patient portal user interface may not be user friendly and make it more difficult for mothers to abide to the study protocol. Another possibility is that online patient portals are still not widely used and accepted by our population, which is supported by several studies analyzing patient portal usage in the United States [17, 18].

We offer several suggestions to improve patient portal use in the clinical realm and studies with newborns. Enrolling newborns before discharge from the hospital or creating a more streamlined enrollment process would likely improve patient portal registration rates. We also suggest efforts must be made to ensure that the patient portal is user friendly, usable by those with low health literacy, and fully smartphone compatible. Despite the challenges we encountered, we encourage future efforts to assess whether patient portals can be used to individualize safe infant sleep care particularly once patients become more accustomed to this technology. Efforts to study differences in patient portal usage among parents of newborns may provide further insight into improving patient portal access and use among this population.

We acknowledge the limitations of this study. Our sample size was lower than anticipated, which results in lower power for our hypothesized effect size. Of those randomized, our cohort was largely non-Hispanic, White, and college educated, with a low proportion of low-income families. Thus, our results may not be generalizable to other demographic groups, and are not generalizable to those patients at highest risk of SUID. Furthermore, participants from lower socioeconomic backgrounds may have a more difficult time accessing internet services, despite indicating that they had full access to internet when enrolling. The lack of photographs with bedsharing may indicate parental unwillingness to share this particular infant care practice or simply the above-mentioned limited statistical power of the study. Lastly, though the presence of wedges underneath or above the mattress was part of the review process, we could not fully assess whether infants were positioned in an inclined versus supine sleep position, as the latter is advised by the AAP.

The current study also occurred within a single health system in Pennsylvania. Given that there are specific state laws for SUID education for parents prior to leaving the hospital, our findings may not be generalizable to other health systems within and outside of Pennsylvania. Parents were aware that their photographs were to be analyzed for safe infant sleep. Thus, it is possible that we may have underestimated the numbers of newborns who were placed in unsafe sleep environments. Nonetheless, our results suggest that parents who sent in photographs demonstrated significant non-adherence to safe infant sleep recommendations.

## Conclusion

Utilizing the EHR to personalize infant sleep recommendations based on photographs submitted by mothers through patient portals is possible, however, we encountered several barriers, including poor patient portal registration and poor response to requests for photographs at study time points. Submitted photographs of infants aged 1–2 months old demonstrated substantial non-adherence to AAP guidelines. Further research is indicated to determine if using the EHR to promote personalized infant sleep safety is effective, and what types of education and interventions are effective at improving safe infant sleep.

## Data Availability

The datasets generated and/or analyzed during the current study are not publicly available due to photographs being submitted through the electronic health record, which are not sharable. However, the remainder of the study data are available from the corresponding author on reasonable request.

## Abbreviations

EHR: Electronic health record
AAP: American Academy of Pediatrics
SUID: Sudden Unexpected Infant Death
SIDS: Sudden Infant Death Syndrome
IQR: Interquartile range
