# Correction to: Improving infant sleep safety via electronic health record communication: a randomized controlled trial

**DOI:** 10.1186/s12887-020-02397-y

**Published:** 2020-10-24

**Authors:** Ethan A. Canty, Benjamin N. Fogel, Erich K. Batra, Eric W. Schaefer, Jessica S. Beiler, Ian M. Paul

**Affiliations:** 1 Department of Pediatrics, Oregon Health & Science University, Portland, OR USA; 2 Department of Pediatrics, Penn State College of Medicine, HS83, 500 University Drive, Hershey, PA 17033 USA; 3 Family and Community Medicine, Penn State College of Medicine, Hershey, PA USA; 4 Public Health Sciences, Penn State College of Medicine, Hershey, PA USA

**Correction to: BMC Pediatr (2020) 20: 468**

**https://doi.org/10.1186/s12887-020-02369-2**

Following the publication of the original article [1], authors spotted mistake in the Funding section. The funding source captured was the Ashley K. Shellenberger SIDS Research Fun, when it should have been the Ashley Nicole Shellenberger SIDS Research Fund. Updated funding section is provided below.

The original article has been corrected.
